# An Adaptive Mapping Method Using Spectral Envelope Approach for DNA Spectral Analysis

**DOI:** 10.3390/e24070978

**Published:** 2022-07-15

**Authors:** Milena Arruda, Andresso da Silva, Francisco de Assis

**Affiliations:** Department of Electrical Engineering, Federal University of Campina Grande, Campina Grande 58428-830, Paraíba, Brazil; andresso.silva@ee.ufcg.edu.br (A.d.S.); fmarcos@dee.ufcg.edu.br (F.d.A.)

**Keywords:** DNA periodicities, genomic signal processing, spectral envelope, spectral analysis, symbolic sequence, three-base periodicity

## Abstract

The digital signal processing approaches were investigated as a preliminary indicator for discriminating between the protein coding and non-coding regions of DNA. This is because a three-base periodicity (TBP) has already been proven to exist in protein-coding regions arising from the length of codons (three nucleic acids). This demonstrates that there is a prominent peak in the energy spectrum of a DNA coding sequence at frequency $\frac{1}{3}$ rad/sample. However, because DNA sequences are symbolic sequences, these should be mapped into one or more signals such that the hidden information is highlighted. We propose, therefore, two new algorithms for computing adaptive mappings and, by using them, finding periodicities. Both such algorithms are based on the spectral envelope approach. This adaptive approach is essentially important since a single mapping for any DNA sequence may ignore its intrinsic properties. Finally, the improved performance of the new methods is verified by using them with synthetic and real DNA sequences as compared to the classical methods, especially the minimum entropy mapping (MEM) spectrum, which is also an adaptive method. We demonstrated that our method is both more accurate and more responsive than all its counterparts. This is especially important in this application since it reduces the risks of a coding sequence being missed.

## 1. Introduction

**Figure 1:**
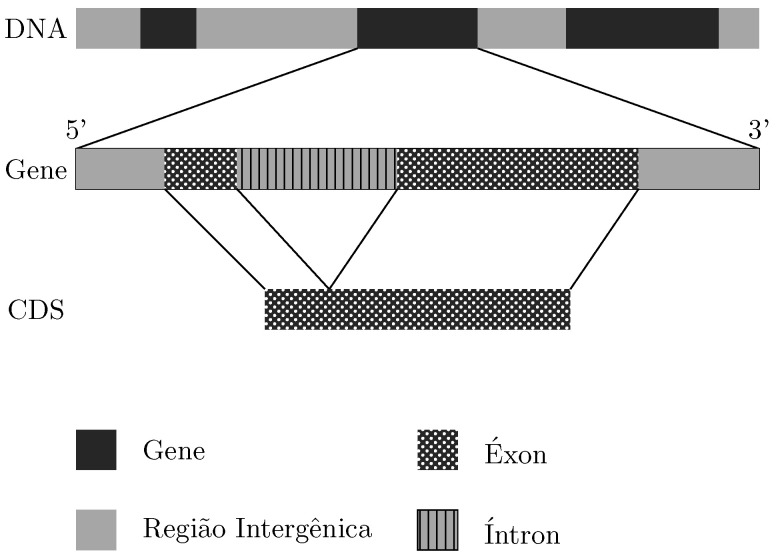
The eukaryotic DNA consists of gene and intergenic regions. Moreover, the gene is composed of regions called exons and introns, which are interleaved with each other.

The development of biological databases and the need to understand how many components present in a living cell are working together to perform cellular functions do justify the growing interest in mathematical, statistical, information theory and computational tools for the analysis of genomic data. In short, the genetic information of an organism is encoded in DNA molecules through units called bases, such as adenine (A), cytosine (C), guanine (G) and thymine (T). In eukaryotic cells, DNA is divided into gene and intergenic regions. The genes are divided into exons and introns. The protein-coding sequences are then the portion of a gene that encodes a protein: its exons. The coding region of a gene is also known as the coding sequence (CDS). The non-coding sequences refer to the introns and intergenic regions (see Figure 1).

In this paper, we investigate how to improve the discrimination between coding and non-coding regions of a DNA sequence. In this sense, Trifonov and Sussman [1] observed the existence of periodicities in DNA sequences from the analysis of the autocorrelation function; Tsonis et al. [2] found that, whereas non-coding regions show a rather random pattern, coding sequences reveal periodicities, in particular, a three-base periodicity (TBP). The TBP property reveals a spectral peak at frequency 13 rad/sample for coding sequences. This periodic phenomenon has attracted the attention of many biologists who are trying to understand and explain it [3,4,5,6]. Thus, it is possible to discriminate between coding and non-coding regions of a DNA sequence by observing its energy spectrum [7,8,9,10].

DNA sequences are symbolic sequences, and, therefore, for spectral analysis, a numerical representation of DNA is first necessary. For that reason, the proper choice of a mapping rule of a DNA sequence onto one or more signals of complex or real numbers must be made. For a given DNA sequence, in particular, mapping is a rule that associates each element of the set of bases N={A,C,G,T}, with an element of another set, such as the set of complex numbers. Consequently, the challenge of predict DNA periodicities is how to choose the mapping rule for such sequences.

A classical method was proposed by Voss [11], in which each of the four bases is associated with a binary indicator signal. Each binary indicator is a discrete-time signal that assumes 1 when the *n*-th symbol of the sequence is a given base and 0 otherwise. Finally, the energy density spectrum is the sum of the energy contribution of each binary indicator signal evaluated from the discrete Fourier transform (DFT) of each signal. In addition, other approaches have proposed mappings from a DNA sequence to a single signal. In this case, the energy spectrum is evaluated from the DFT of this signal. Among the most common mappings, Nair and Sreenadhan [12] proposed a mapping based on the electron-ion interaction pseudopotentials (EIIP); Anastassiou [13] proposed that in the mapping image were complex numbers, similar to the QPSK modulation technique; and Galleani and Garello [14] proposed the minimum entropy mapping (MEM) spectrum, in which a real mapping is computed from the spectral entropy minimization criterion.

However, these approaches have some performance limitations. The most important limitation is how to define this mapping. Symbolic sequences have a statistical structure that provides important information about them. We, therefore, expect that a numerical representation of such sequence does not impose additional features on the resulting signal. For example, a map cannot assume that one symbol is always numerically greater than another. For this reason, it is clear that the same mapping for any DNA sequence must ignore the features that are particularly inherent to it. Thus, this suggests that for each DNA sequence, a particular mapping should be performed.

Assuming that a numerical signal is appropriate for a given DNA sequence, then time–frequency analysis can be applied to detect coding regions in genes. In this sense, Tiwari et al. [15] were the first researchers to propose that it is sufficient to evaluate the energy density at frequency 13 rad/sample in a window of *W* samples, sliding it through the set of binary indicators. Vaidyanathan and Yoon [16] proposed the use of the antinotch filter on the sliding window over the set of binary indicators. Sahu and Panda [17] suggested the use of the S transform, considering the signal resulting from the EIIP mapping. Wang and Johnson [18] expanded the spectral envelope approach (initially proposed by Stoffer et al. [19]) to processing non-stationary symbolic signals in the time–frequency domain and analyzed the correlation structure of DNA.

Therefore, in this paper, we propose two new algorithms for computing mappings for DNA sequences. Both algorithms are based on the spectral envelope approach. Briefly, the spectral envelope is the new spectrum obtained by maximizing the energy spectrum over the entire frequency range [0, N − 1]. That is, at each frequency in this range, the spectral envelope looks for four constants on a complex hypersphere with a unit radius that maximizes the energy density spectrum of the signal resulting from the linear combination of the binary indicator signals. Note that N+1 combinations are computed, each of which is a potential DNA mapping.

We then use this mapping to find the numerical signal for the DNA sequence. Thus, we can calculate the respective energy density spectrum of the signal to discriminate between coding and non-coding sequences. The first algorithm searches for the mapping that maximizes the SNR of the energy density spectrum. The second algorithm, on the other hand, takes advantage of prior knowledge about the TBP property such that the mapping results from the spectral envelope at the frequency k=⌊N3⌋.

The performance of the new methods is verified by comparing it with the performance of four other well-established methods in the literature—Voss [11], EIIP [12], QPSK [13] and MEM spectrum [14]—and by applying them to synthetic and real DNA sequences whose properties are known. In addition, we make remarks about the proposed algorithms by discussing their intrinsic properties and computational complexities. Finally, the use of our methods shows results that have outperformed the discrimination of TBP in DNA sequences in contrast with previous works. Moreover, we noticed improvements in the SNR and spectral entropy of the respective signals. The algorithms were implemented in Python and are available in the following GitHub repository [20].

The present paper is organized as follows. Section 2 provides notations and definitions that are important to the analyses in this paper. In Section 3, we present our methods and the proposed algorithms. In Section 4, we make remarks on the algorithms. The results are presented and discussed in Section 5 and, finally, the conclusions are elaborated in Section 6.

## 2. Preliminaries

In this section, we describe some notation and definitions that are important to the analyses in this paper; for more details, we recommend [21,22]. Let v denote a *n*-dimensional vector characterized by its *n* components, i.e., $v=[v_{1}\;\;v_{2}\;\;\ldots \;\;v_{n}]$. The norm of this vector is denoted by ||v|| and is defined as

(1) $$\left||v|\right|=\sqrt{\sum_{i=1}^{n}{\left|v_{i}\right|}^{2}}.$$

Due to the fact that discrete-time signals have the same basic properties of vectors, a signal x[n], for example, defined on some interval [0,N[ is represented in vector form as $x=[x_{0}\;\;x_{1}\;\;\ldots \;\;x_{N-1}]$. Thus, the norm can also be evaluated for signals.

### 2.1. DNA Numerical Representation

Let *s* be a given DNA sequence of length *N*. A mapping M is defined as the association between the four DNA bases and four complex numbers. That is,

(2) M:A↦a,C↦c,G↦g,T↦t,

such that its image is given by

(3) a,c,g,t∈C.

**Figure 2:**
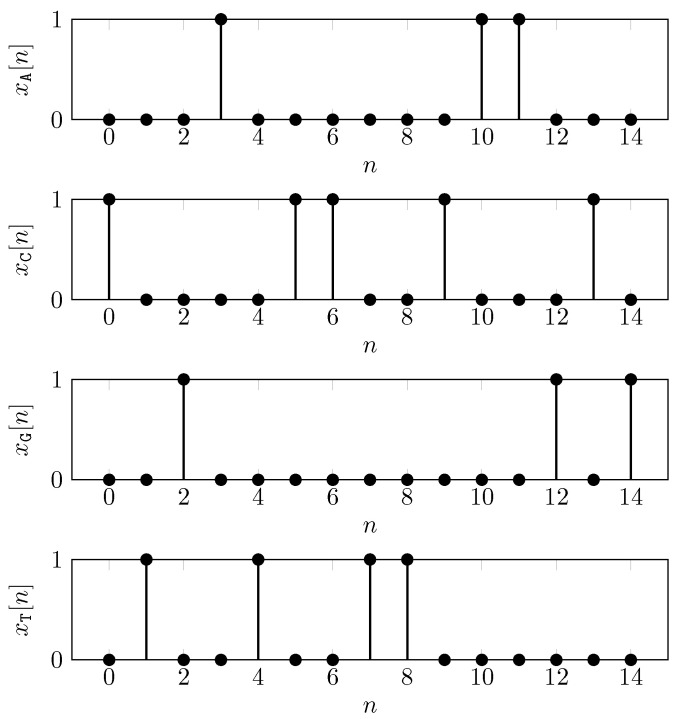
Binary indicator functions for s=CTGATCCTTCAAGCG.

Supposing that the first four nucleotides of a given DNA sequence are s=ACGT⋯, we can, therefore, associate the following discrete-time signal to *s* by using the mapping M,

(4) x[n]=aδ[n]+cδ[n−1]+gδ[n−2]+tδ[n−3]+⋯,

where δ[n] is the unit impulse function. An alternative form to (4) is

(5) $$x\left[n\right]=ax_{A}\left[n\right]+cx_{C}\left[n\right]+gx_{G}\left[n\right]+tx_{T}\left[n\right],$$

where $x_{\alpha }\left[n\right]$ is the binary indicator signal. The binary indicator signal assumes 1 when the *n*-th symbol in *s* is the basis α∈{A,C,G,T}, and 0 otherwise. For example, the binary indicator functions for the sequence s=CTGATCCTTCAAGCG are shown in Figure 2 and their representation in vector form is as follows,

$$\begin{array}{cc} x_{A} & =\left[0\;\;0\;\;0\;\;1\;\;0\;\;0\;\;0\;\;0\;\;0\;\;0\;\;1\;\;1\;\;0\;\;0\;\;0\right],\\ x_{C} & =\left[1\;\;0\;\;0\;\;0\;\;0\;\;1\;\;1\;\;0\;\;0\;\;1\;\;0\;\;0\;\;0\;\;1\;\;0\right],\\ x_{G} & =\left[0\;\;0\;\;1\;\;0\;\;0\;\;0\;\;0\;\;0\;\;0\;\;0\;\;0\;\;0\;\;1\;\;0\;\;1\right],\\ x_{T} & =\left[0\;\;1\;\;0\;\;0\;\;1\;\;0\;\;0\;\;1\;\;1\;\;0\;\;0\;\;0\;\;0\;\;0\;\;0\right]. \end{array}$$

An alternative form to (5) is to define it in vector form as follows,

(6) $$\begin{array}{cc} x & =ax_{A}+cx_{C}+gx_{G}+tx_{T}\\ \; & =w\left[\begin{array}{c} x_{A}\\ x_{C}\\ x_{G}\\ x_{T} \end{array}\right], \end{array}$$

where $w=\left[a\;\;c\;\;g\;\;t\right]$ are the elements of M called the weight vector. Each binary indicator vector is *N*-dimensional and x is also a *N*-dimensional.

The signal x[n] will be a real or complex-valued signal depending on whether the mapping M is also real or complex, respectively. The most common complex mapping is the QPSK mapping [13] given by

(7) M:A↦1+j,C↦−1−j,G↦−1+j,T↦1−j.

The most common real mapping is the electron-ion interaction pseudopotentials (EIIP) indicator proposed by Nair and Sreenadhan [12]. This is given by

(8) M:A↦0.126,C↦0.134,G↦0.0806,T↦0.1335.

Additionally, Galleani and Garello [14] proposed the real mapping called MEM spectrum, which is based on the criterion of entropy minimization of the energy spectrum.

### 2.2. Spectral Analysis Overview

The spectral analysis is performed on the signals resulting from the numerical representation of DNA to find periodicities in genomic sequences. The classical approach was particularly proposed by Voss [11]. Here, the energy spectral density of a given DNA sequence *s* is the sum of the energy contribution of their binary indicator functions, as follows:

(9) $$S_{\mathrm{Voss}}\left[k\right]=|X_{A}\left[k\right]{|}^{2}+|X_{C}\left[k\right]{|}^{2}+|X_{G}\left[k\right]{|}^{2}+|X_{T}\left[k\right]{|}^{2},$$

where $X_{\alpha }\left[k\right]$ with α∈{A,C,G,T} is the discrete Fourier transform (DFT) of the respective binary indicators, that is,

(10) $$X_{\alpha }\left[k\right]=\sum_{n=0}^{N-1}x_{\alpha }\left[n\right]e^{-j\frac{2\pi }{N}nk},\;\;\;k=0,1,\ldots ,N-1.$$

Since a given DNA sequence *s* is mapped to a signal in the form of (5), its energy spectrum is given by

(11) $$S\left[k\right]={\left|aX_{A}\left[k\right]+cX_{C}\left[k\right]+gX_{G}\left[k\right]+tX_{T}\left[k\right]\right|}^{2}.$$

The energy spectrum can be symmetric or asymmetric on the frequency axis, depending on whether the mapping is real or complex. For this reason, it is important to compute carefully the one-sided spectrum by adding the spectral content at negative frequencies with the spectral content at positive frequencies.

An alternative form to (11) is to define it in vector form. Let $X_{\alpha }^{k}$ be the DFT coefficient of binary indicator signals of the base α at a particular frequency *k*, and we define the 4-dimensional vector $X_{k}$ as

(12) $$X_{k}=\left[X_{A}^{k}\;\;X_{C}^{k}\;\;X_{G}^{k}\;\;X_{T}^{k}\right],$$

so, the energy spectrum at a particular frequency *k* is given by

(13) $$S_{k}=wX_{k}^{*}X_{k}w^{T},\;\;\;k=0,1,\ldots ,N-1,$$

where $w^{T}$ is the transpose of $w=\left[a\;\;c\;\;g\;\;t\right]$, $X_{k}^{*}$ is the conjugate transpose of $X_{k}$ and $S_{k}$ is a scalar. Therefore, the vector form for the energy spectrum is given by

(14) $$S=[S_{0}\;\;S_{1}\;\;\cdots \;\;S_{N-1}],$$

where S is a *N*-dimensional vector.

### 2.3. Spectral Entropy

Analogous to Shannon entropy, spectral entropy characterizes the irregularity of the energy distribution in the frequency domain. It will be used as a comparative measure of uncertainty of the periodicities. It is defined [23] as

(15) $$H\left(S\left[k\right]\right)=-\sum_{k=0}^{\left\lfloor N/2\right\rfloor }p\left[k\right]\log p\left[k\right],$$

where $p\left[k\right]=\frac{S[k]}{\sum_{k=0}^{\left\lfloor N/2\right\rfloor }S\left[k\right]}$. In this paper, the natural logarithm is used in (15), so the spectral entropy is given in nats. A signal whose energy is approximately equally distributed over the frequencies has maximal spectral entropy, and the spectrum of a single frequency signal has minimal spectral entropy, which is zero. The minimum value of H(S[k]) is zero and occurs when p[k]=1 for some *k*. The maximum value occurs when the energy distribution is uniform, in this case, H(S[k])=log⌊N2⌋+1 nats.

### 2.4. Spectral Envelope

As we have seen, the spectral envelope is the new spectrum of a given signal obtained by maximizing the energy spectrum over the entire frequency range $\left[0,N-1\right]$. That is, the spectral envelope of a signal, such as (6), is defined at a particular frequency *k* as the maximum spectrum subject to all possible non-trivial weight vectors and regularized by ||w||=1. Note that the components of w are complex numbers.

Therefore, for each frequency *k* the spectral envelope [18,19] is given by

(16) $$\lambda_{k}=\max_{\begin{array}{c} w\in C^{4}\\ ||w||=1 \end{array}}S_{k}=\max_{\begin{array}{c} w\in C^{4}\\ ||w||=1 \end{array}}wX_{k}^{*}X_{k}w^{T},$$

where $X_{k}^{*}X_{k}$ is a Hermitian matrix.

This maximization operation is the same as maximizing the Rayleigh quotient. The Rayleigh quotient is maximized when w is the eigenvector corresponding to the largest eigenvalue of $X_{k}^{*}X_{k}$. Thus, assume that the eigendecomposition of this squared matrix is of the form

(17) $$X_{k}^{*}X_{k}=Q\Lambda Q^{-1},$$

where Q is a square 4×4 matrix whose *i*th column is the eigenvector $q_{i}$ of $X_{k}^{*}X_{k}$, and Λ is a diagonal matrix whose diagonal elements are the corresponding eigenvalues, $\Lambda_{ii}=\lambda_{i}$. So, the spectral envelope is the largest eigenvalue, that is,

(18) $$\lambda_{k}=\max_{i\in 1,2,3,4}\lambda_{i},$$

when w is its corresponding eigenvector. The pseudocode on how to determine $\lambda_{k}$ and w in (16) is shown in Algorithm 1.
**Algorithm 1**SpectralEnvelope (*s*, *k*)**Input:** DNA sequence *s* and frequency *k* **Output:** Mapping M
 1:Compute the set of binary indicator vectors of *s* 2:Compute $X_{k}$ using (10) and (12) 3:Eigendecomposition of $X_{k}^{*}X_{k}\leftarrow Q\Lambda Q^{-1}$ 4:$\lambda_{k}\leftarrow \max\left(\mathrm{diag}\left(\Lambda \right)\right)$ 5:w← eigenvector of $\lambda_{k}$ 6:**return** w


## 3. Methods

### 3.1. Experimental Data

The data are available at the nucleotide database from the National Center for Biotechnology Information (NCBI) that provides open access to biomedical and genomic information [24]. Each DNA sequence record processed by NCBI is referred to by an accession number. Furthermore, the qualifier that links DNA sequence records and their genes is the geneID. The accession numbers and geneID are both a simple series of digits.

For a detailed analysis of spectrum methods, we use the chromosomes XIV, XV, and XVI of *Saccharomyces cerevisiae* (accession numbers NC_001146.8, NC_001147.6 and NC_001148.4, respectively). Each chromosome has 398, 546, and 474 coding sequences, respectively. For the coding sequence whose orientation is complementary, we perform the complement reverse operation to start each sequence at the codon ATG. The data are divided into two datasets: the first has only coding sequences (the coding sequence dataset) and the second has only sequences from intergenic regions (the non-coding sequence dataset). In both cases, we discard sequences whose length is less than 200 base pair (bp). Finally, there are 1388 coding sequences and 1188 non-coding sequences in our dataset.

Furthermore, we use the portion of gene *F56F11* from chromosome III of *Caenorhabditis elegans* that transcribes the protein *F56F11.4*, *isoform a*. The *F56F11.4a* is used as a benchmark problem for different exon detection techniques [8,13,14]. It has 7990 bp starting at nucleotide position 7021 of gene *F56F11*. In addition, the *F56F11.4a* has five well-known distinct exons whose locations relative to nucleotide position 7021 vary from 928 to 1039, 2528 to 2857, 4114 to 4377, 5465 to 5644 and 7255 to 7605. Note that the first exon is the shortest (112 bp) and usually the most difficult to detect.

### 3.2. Adaptive DNA Mappings

As we have seen previously, the first procedure for the spectral analysis of DNA sequences is mapping the symbolic data to a numeric signal. We have also seen that having a single mapping for all DNA sequences can ignore the intrinsic properties of each sequence. Therefore, an adaptive mapping should be done by searching potential mappings for DNA sequences in order to highlight the structure of their data. To implement the adaptive mapping method, we propose the use of a spectral envelope approach.

One should recall that the envelope spectral represents the maximum energy that the signal (6) can have such that ||w||=1. For each particular frequency in the entire range $k\in \left[0,N-1\right]$, there is a respective w. These vectors are the search space for our adaptive DNA mapping method based on the spectral envelope. For each w, there is an associated mapping M. The image of a particular mapping M, that is, a,c,g,t∈C, are the components of a respective vector w. Therefore, in our search space, there are up to *N* potential mappings and *N* different signals, which can also differ in their spectral composition.

**Figure 3:**
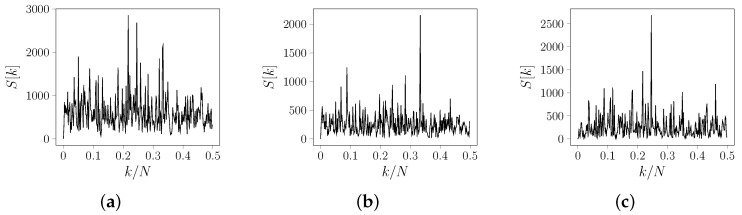
Spectral analysis for the CDS of gene *AIM41* (geneID: 854390) from chromosome XV of *Saccharomyces cerevisiae* where N=558. (**a**) Spectral envelope. (**b**) Energy spectrum of the signal mapped by using $M_{1}$ as in (19). (**c**) Energy spectrum of the signal mapped by using $M_{2}$ as in (20).

For example, consider the coding sequence of the *AIM41* gene (geneID: 854425) from chromosome XV of *Saccharomyces cerevisiae*. Since it is a coding sequence, we expect the presence of the TBP property, and, therefore, we expect its energy spectrum to reveal a discriminant spectral peak at frequency 13 rad/sample. The spectral envelope for this sequence is shown in Figure 3a. Note that, instead of what is expected by the TBP property for the spectral envelope, the peak occurs at the k=0.22 rad/sample. However, when we solve the spectral envelope at the frequency k1=13 rad/sample, we obtain $\lambda_{k_{1}}=2157.64$ and $w_{k_{1}}=\left[0.43\;\;-0.25+0.29j\;\;-0.13-0.69j\;\;-0.05+0.41j\right]$. Therefore, the corresponding mapping is given by

(19) $$\begin{array}{cc} M_{1}:\; & A\mapsto 0.43,\;C\mapsto -0.25+0.29j,\\ \; & G\mapsto -0.13-0.69j,\;T\mapsto -0.05+0.41j. \end{array}$$

The energy density spectrum of the signal, mapped by using $M_{1}$, is shown in Figure 3b. In this case, as expected from the TBP property, the peak occurs at k=0.33 rad/sample. However, the TBP property is not observed for all w in the search space. At frequency $k_{2}=0.24$ rad/sample, the spectral envelope is $\lambda_{k_{2}}=2687.59$, and the corresponding mapping is given by

(20) $$\begin{array}{cc} M_{2}:\; & A\mapsto 0.41,\;C\mapsto 0.48+0.23j,\\ \; & G\mapsto -0.29-0.07j,\;T\mapsto -0.06+0.29j. \end{array}$$

The energy density spectrum of the signal, mapped by using $M_{2}$, is shown in Figure 3c. Notice that the energy spectrum of the DNA sequences can be slightly different when we change the mapping. This can be another reason to look for adaptive and unique mappings for each sequence.

To select a single mapping for a DNA sequence, we must choose it from the *N* potential mappings. For this reason, a constraint should be imposed. The first algorithm uses as the constraint the maximization of SNR of the energy density spectrum. Consequently, from now on, we will call it SNR-SE, where SE is the short form for the spectral envelope. The SNR is the ratio of signal power to noise power. It is computed on the energy density spectrum of the signal as follows. The signal power is estimated as the energy of the highest spectral component; the noise power or the background noise is the total energy, excluding the signal power and the DC value [4].

In this algorithm, the potential mappings are those that solve the spectral envelope for each frequency *k* in the closed interval from 0 to ⌊N2⌋. The search space is reduced since the one-sided energy spectrum must have all the spectral information about the signal. Therefore, for each potential mapping, the energy spectrum and its SNR are estimated. Finally, we choose the mapping whose respective signal has the energy spectrum with the highest SNR. The pseudocode of this method is shown in Algorithm 2.

The second algorithm is but a special case of the first. Now we will exploit previous knowledge of the TBP property. As a result, from now on, we will call it TBP-SE. We assume that all coding sequences have the TBP property, so a discriminant spectral peak at frequency 13 rad/sample is observed, whereas, in non-coding sequences, this peak is absent. Therefore, from among all potential mappings of the spectral envelope, this algorithm chooses the one that solves the optimization problem of the spectral envelope at frequency k=⌊N3⌋ rad/sample. The pseudocode of this method is shown in Algorithm 3.
**Algorithm 2**SNR-SE**Input:** DNA sequence *s* **Output:** One sided spectrum S[k] 1:$snr_{ref}\leftarrow -\infty$ 2:**foreach***k***in**[0,⌊N2⌋]**do** 3:   w←SpectralEnvelope (*s*, *k*) 4:   $M_{ref}\leftarrow$ map whose image are the components of w 5:   Compute S[k] using Equation (11) 6:   snr←SNR(S[k]) 7:   **if** $snr_{ref}<snr$ **then** 8:       $snr_{ref}\leftarrow snr$ 9:       $M\leftarrow M_{ref}$10:   **end if**11:**end for**12:Compute S[k] using (11) and M13:**return** S[k]
**Algorithm 3**TBP-SE**Input:** DNA sequence *s* **Output:** One sided spectrum S[k] 1:k←⌊N3⌋ 2:w←SpectralEnvelope (*s*, *k*) 3:M← map whose image are the components of w 4:Compute S[k] using (11) and M 5:**return** S[k]

### 3.3. Evaluation and Interpretation

The spectral analysis for the discrimination of the DNA coding sequences is then evaluated as follows. We must check at which frequency the largest spectral peak occurs. If it occurs between frequencies 13±0.02 rad/sample, we say that such a sequence is a DNA coding sequence.

Therefore, the test outcome can be positive (classifying the DNA sequence as a coding sequence) or negative (classifying the DNA sequence as a non-coding sequence). The test results for each DNA sequence may or may not match the real status. In such a setting, we have the following:

- True positive: coding sequences that are correctly identified as coding sequences;
- False positive: coding sequences that are misclassified as non-coding sequences;
- True negative: non-coding sequences that are correctly classified as non-coding sequences;
- False negative: non-coding sequences that are misclassified as coding sequences.

To compare the effectiveness of each DNA coding sequence identification method, we evaluate three measures: accuracy, sensitivity, and specificity. Accuracy defines the global correct classification rate, reflecting the ability to predict correctly concerning total samples, that is,

(21) $$accuracy=\frac{\mathrm{TP}+\mathrm{TN}}{\mathrm{TP}+\mathrm{TN}+\mathrm{FP}+\mathrm{FN}}.$$

Sensitivity or true positive rate (TPR) evaluates the ability to correctly predict a coding sequence, that is,

(22) $$\mathrm{TPR}=\frac{\mathrm{TP}}{\mathrm{TP}+\mathrm{FN}}.$$

Specificity or true negative rate (TNR) evaluates the ability to correctly predict a non-coding sequence, that is,

(23) $$\mathrm{TNR}=\frac{\mathrm{TN}}{\mathrm{TN}+\mathrm{FP}}.$$

If these tests show that the sensitivity is high, then any DNA sequence that is a coding sequence is likely to be classified as a coding sequence by the method. On the other hand, if the specificity is high, any DNA sequence, which is a non-coding sequence, is likely to be classified as a non-coding sequence by the test. The best possible prediction method would yield the following result: 100% sensitivity (no false negatives) and 100% specificity (no false positives).

## 4. Remarks on Algorithm

### 4.1. Complex Mapping

Since the proposed algorithms search for complex mappings, the energy density spectrum can be asymmetric on the frequency axis. For this reason, it is extremely important to take into account the content of both positive and negative frequencies. Let us consider, for example, the periodic sequence (with 3 bp and 6 bp periodicities) of which we show the first period only,

(24) s=CACCCG⋯.

In fact, we can see that on the following sinusoid signal with the same discrete periodicities,

(25) $$x\left[n\right]=\sin\left(\frac{2\pi }{3}n\right)+\sin\left(\frac{2\pi }{6}n\right).$$

The signal x[n] takes only three values $-\sqrt{3}$, 0, and $\sqrt{3}$. If we define $M:A\mapsto \sqrt{3}$, C↦0, $G\mapsto -\sqrt{3}$ and T↦t where *t* can assume any value, the resulting signal of mapping *s* by using M is (25).

**Figure 4:**
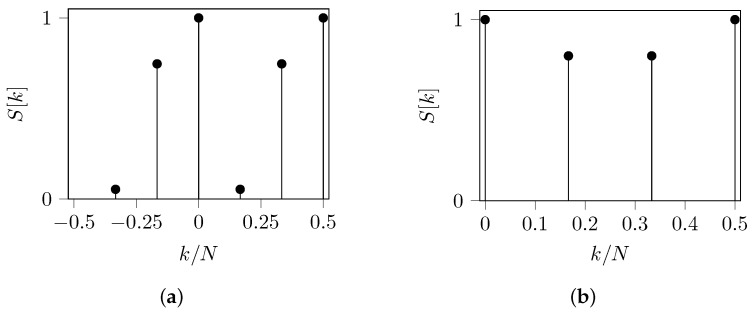
QPSK energy density spectrum of the sequence *s* defined in (24). (**a**) Two-sided spectrum: there are two peaks at frequencies k1=16 rad/sample and k2=13 rad/sample, but with different content. (**b**) One-sided spectrum: there are two peaks at frequencies k1=16 rad/sample and k2=13 rad/sample with the same content.

Therefore, we expect the energy density spectrum of *s* to have two peaks at frequencies k1=16 rad/sample and k2=13 rad/sample. In Figure 4a, we reveal the QPSK energy density spectrum of *s* as being asymmetric on the frequency axis and having two other peaks at k=0 and k=12. Since these peaks are traditionally discarded in the spectral analysis, we focus only on the other peaks. Note that if we analyze only the content of positive frequencies, the peak at $k_{1}$ is smaller than the peak at $k_{2}$; and if we analyze only the content of negative frequencies, the peak at $-k_{1}$ is greater than the peak at $-k_{2}$; nonetheless both peaks should have the same content. For a reliable analysis, the one-sided spectrum must be computed, adding the spectral content at negative frequencies to the spectral content at the positive frequencies. Finally, the one-sided spectrum of *s*, using the QPSK mapping, is shown in Figure 4b. Here, both peaks have the same content.

For this specific *s*, our algorithms have also found a complex mapping. The TBP-SE found M:A↦0.58, C↦−0.29−0.5j, G↦−0.29+0.5j and T↦0; and the SNR-SE found M:A↦0.50, C↦−0.71+0.2j, G↦−0.45−0.004j and T↦0. Both algorithms produce the same energy density spectrum as shown in Figure 4. A similar analysis should be performed for any other DNA sequences and complex mappings.

### 4.2. Adaptive Mappings

Symbolic sequences can have a statistical structure that provides important information about them. Therefore, a mapping from the symbolic to the numeric domain will be required to avoid additional features to occur in the symbolic sequence beyond that which is inherent to it. For example, an arbitrary mapping would be to assign the alphabetically sorted nucleotides to an increasing sequence of integers, as follows, M:A↦1,C↦2,G↦3 and T↦4. However, this M suggests that one nucleotide is somehow greater than another, which is a property that this symbolic set does not have [18].

Another example is the periodic sequence, whose the first period is shown by

(26) s=ACGTGC⋯.

**Figure 5:**
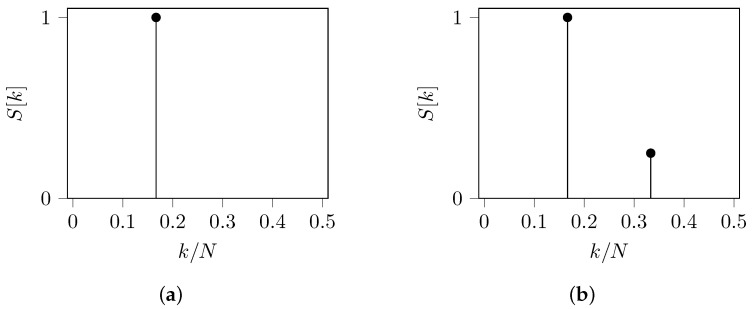
One-sided energy density spectrum of the sequence *s* defined in (26) when: (**a**) $M_{1}:A\mapsto 1$, C↦0.5, G↦−0.5 and T↦−1 is used; and (**b**) $M_{2}:A\mapsto 1.5$, C↦0.25, G↦−0.75 and T↦−0.5 is used.

The energy density spectrum of *s* changes significantly depending on the mapping used. For example, when $M_{1}:A\mapsto 1$, C↦0.5, G↦−0.5 and T↦−1, then S[k] has only one peak at k=16 rad/sample (see Figure 5a). However, if $M_{2}:A\mapsto 1.5$, C↦0.25, G↦−0.75 and T↦−0.5, then S[k] has a peak at k=16 rad/sample and a smaller peak at k=13 rad/sample (see Figure 5b). Notice that, depending on the mapping, we can detect or not an additional periodicity at frequency 13 rad/sample. In this case, the same sequence was mapped to different signals that did not always reveal all spectral information of a symbolic sequence. This is another reason to emphasize the importance of mapping flexibility.

In contrast, in our proposed algorithms, the mapping does not act as a parameter and is chosen uniquely for each sequence. This is also the case of the MEM spectrum [14], where the spectral entropy minimization criterion is used. However, the spectral entropy is invariant under the permutation of the power spectrum estimates on the frequency range, thus ignoring the intrinsic partial order structure of a signal [23]. Hence, very different signals in the time domain yield the same spectral entropy, and this optimization criterion may lose information about the signal.

### 4.3. Exploiting the TBP Property

Especially in the applications where we deal with symbolic sequences and there is prior knowledge about their spectral characteristics, we can use such information to improve the analysis. For example, we know that the TBP property is present in exonic regions and absent in intronic regions, so we should check if it is possible to maximize this frequency content to improve the discrimination of DNA regions. This is exactly what is proposed in the TBP-SE algorithm. This statement is supported by the results presented in the next section.

### 4.4. Computational Complexity

The computational complexity of the spectral analysis algorithms discussed in this paper will be discussed in the sense of the big *O* notation. The big *O* notation is particularly useful for studying the worst-case behavior of specific algorithms, where we are often satisfied with an upper bound on the resources consumed by an algorithm [25].

Note that the DFT computation of the four binary indicator functions is the common step for all algorithms. This operation has complexity O(NlogN), where *N* is the sequence length. In some methods, this is the term with the highest order, and therefore we say that Voss [11], EIIP [12], QPSK [13], SNR-SE and TBP-SE are O(NlogN). On the other hand, the additional operations required in MEM Spectrum [14] have quadratic order, so the MEM spectrum is $O(N^{2})$.

## 5. Results and Discussions

The energy density spectrum of DNA sequences can be slightly different when we compare different methods of spectral analysis. In general, these spectrums do not represent approximated versions of the other. For comparison, the energy spectrum of all sequences in the database was evaluated using the two algorithms proposed in this paper: SNR-SE and TBP-SE, in addition to these four methods already consolidated in the literature: Voss [11], EIIP [12], QPSK [13] and MEM spectrum [14].

**Figure 6:**
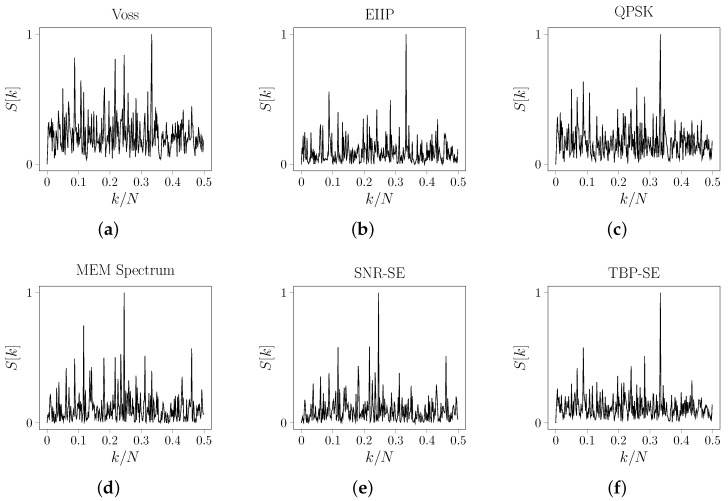
Normalized energy density spectrum for the CDS of gene *AIM41* (geneID: 854390) from chromosome XV of *Saccharomyces cerevisiae* where N=558. (**a**) Voss. (**b**) EIIP. (**c**) QPSK. (**d**) MEM Spectrum. (**e**) SNR-SE. (**f**) TBP-SE.

**Figure 7:**
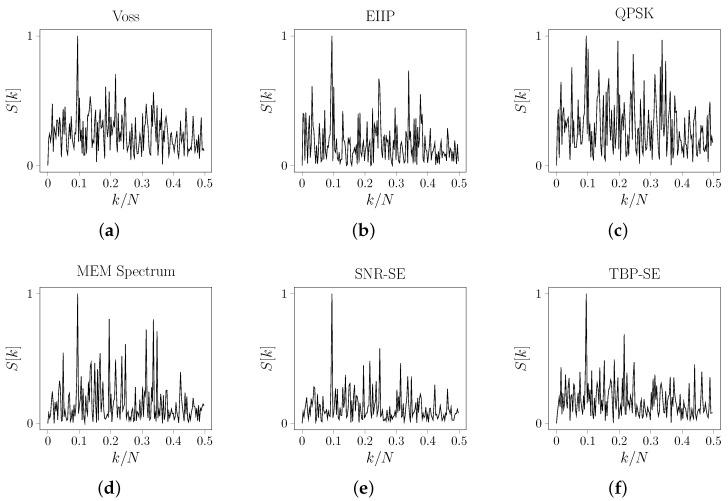
Normalized energy density spectrum for the CDS of gene MRP35 (geneID: 855601) from chromosome XIV of *Saccharomyces cerevisiae* where N=348. (**a**) Voss. (**b**) EIIP. (**c**) QPSK. (**d**) MEM Spectrum. (**e**) SNR-SE. (**f**) TBP-SE.

Consider the specific case of the *AIM41* and *MPR35* genes whose energy spectrums are shown in Figure 6 and Figure 7, respectively. Note that, instead of what is expected, not all methods detect the TBP property for the genes. There are two possible reasons for this. First, the mapping chosen can hide spectral information on the sequence. For the *AIM41* gene, for example, the energy density spectrum, as defined by Voss or using EIIP, QPSK, and TBP-SE mappings, has the largest peak at frequency 0.33 rad/sample. However, observe that the background noise increases significantly when the Voss is evaluated. In addition, this discriminatory frequency is lost when the MEM spectrum and SNR-SE are evaluated (see Figure 6).

The second reason is that, although the TBP property in coding sequences is a classical frequency discriminator in the biological context, some coding sequences do not seem to be distinguished by it. This is the case with the *MPR35* gene. For all methods, the energy density spectrum has the largest peak at the frequency 0.09 rad/sample (see Figure 7). Beyond these cases, in general, the spectrum evaluated by our methods yields improvements in the coding sequence classification and background noise reduction.

Although there are intrinsic limitations in the spectral analysis of a given DNA sequence, some methods can better discriminate the TBP property for coding sequences than others. Table 1 compares all the methods already mentioned regarding the accuracy, sensitivity, and specificity. Note that there is often a trade-off between sensitivity and specificity, such that by increasing sensitivity, one can decrease specificity and vice versa.

Table 1 reveals that our proposed method, TBP-SE, had the highest accuracy and sensitivity among all. This is especially important in this application since we reduce the probability that a coding sequence will not be identified. In other words, coding sequences are more likely to be correctly identified as coding sequences using TBP-SE. Furthermore, the specificity had an expressive level, and TBP-SE had the most uniform levels of accuracy, sensitivity, and specificity.

On the other hand, when comparing the methods with adaptive mapping, MEM spectrum does not perform well. It has the lowest levels of accuracy and sensitivity. One possible reason for this is that the search space of this method is constrained by spectral entropy; nevertheless, spectral entropy ignores the intrinsic structure of partial order, as pointed out by [23]. Furthermore, this method has the highest computational complexity, and it is not feasible when compared to the other spectral analysis methods discussed in this paper.

**Figure 8:**
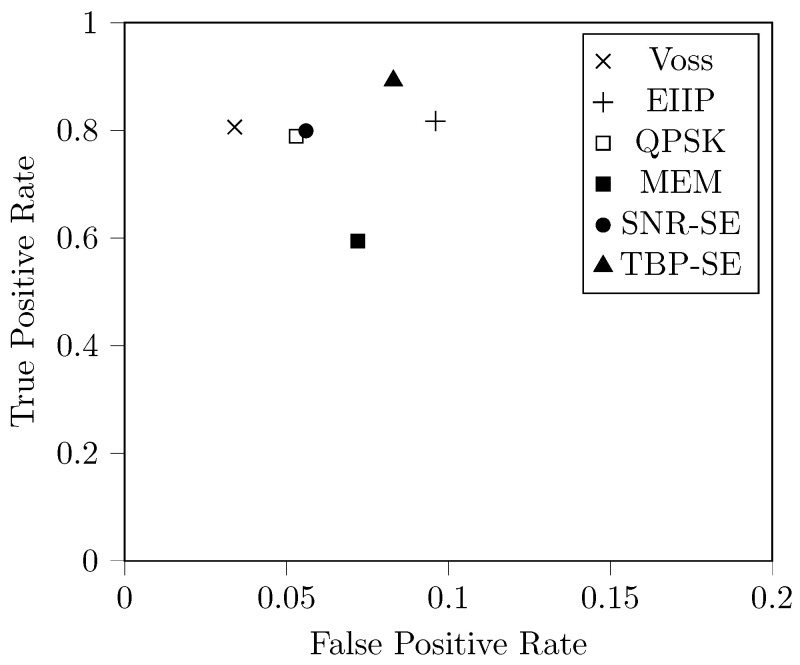
Receiver operating characteristic (ROC) curve for the spectral analysis methods of DNA coding sequences.

The other methods seem to perform similarly to each other, but differences can be noted graphically via the receiver operating characteristic (ROC) curve, see Figure 8. The ideal ROC curve hugs the top left corner, indicating a high TPR and a low False Positive Rate (FPR), where FPR=1−TNR. Since we use a binary classification without a threshold, the method statistics yield a single point on the ROC space.

The ROC curve reveals that QPSK and SNR-SE have similar performance (the difference in TPR is 0.01 and in FPR is 0.003). In addition, Voss, EIIP, QPSK, and SNR-SE have approximately the same TPR, but Voss performs better because it has the lowest FPR. Taking both the Voss and TBP-SE into account, approximately 97.31% of coding sequences that were misclassified as non-coding sequences using TBP-SE were also misclassified using Voss. This phenomenon also occurs in the MRP35 gene (see Figure 7), but still, the background noise of the DNA spectrum is reduced using TBP-SE. Therefore, TBP-SE can be preferred over Voss, since the TPR level is especially important in this application.

### Case Study: Gene F56F11.4a

The gene *F56F11.4a* has five well-known distinct exons whose locations relative to nucleotide position 7021 are between 928 and 1039, 2528 and 2857, 4114 and 4377, 5465 and 5644, and 7255 and 7605. The first exon is the shortest (112 bases) and is usually the most difficult to detect. In this scenario, the coding regions are identified as follows [8,10,15]. The energy density spectrum at frequency 13 rad/sample is evaluated over a window of *W* samples, then the window is slid by one or more samples, and the energy density is recalculated in a process that analyzes the entire DNA sequence. An important criterion for this analysis is to define the window length *W*. For this gene, Tiwari et al. [15] suggests using W=351. Therefore, a rectangular window of length 351 and step size 5 was used.

**Figure 9:**
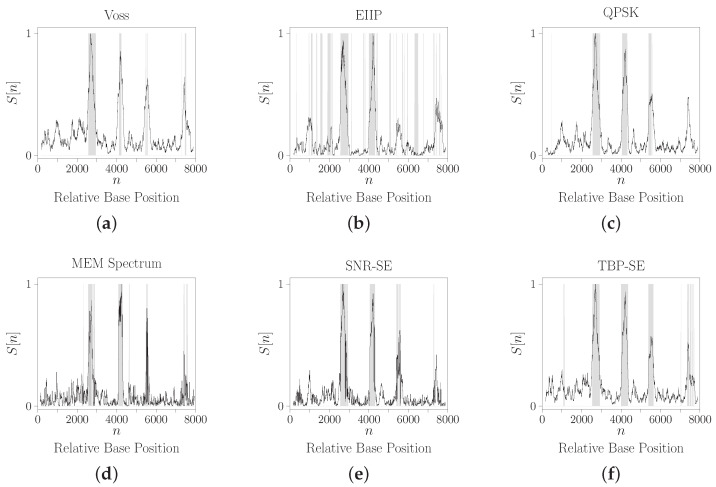
Energy density spectrum of the gene *F56F11.4a* by using window size of W=351 the following methods: (**a**) Voss. (**b**) EIIP. (**c**) QPSK. (**d**) MEM spectrum. (**e**) SNR-SE. (**f**) TBP-SE.

For comparison purposes, the results are presented in Figure 9, where the horizontal axis is the relative base positions and the vertical axis is the energy density spectrum normalized by its maximum value. There are two possible interpretations. First, the peaks in the spectrum should correspond to the regions where the TBP property is present. These regions can be evaluated using a threshold, that is, the coding regions are identified by putting a threshold on the spectrum, so regions having energy above this threshold are considered exons. In this case, in general, the methods detect four of the five exons and the first exon is the most missed. Specific to the EIIP method, the energy of the fourth exon is significantly reduced by mixing it with intronic regions. The other methods have similar performance.

However, the second interpretation expresses more information about the gene. In this case, the shaded areas show the regions where the TBP property is present in the respective slide window. The TBE-SE was the only one to identify the presence of all five exons. The EIIP indeed showed to have more instability in predicting non-coding regions. Voss, QPSK, MEM and SNR-SE had similar performances, but the MEM seems to increase the background noise of the spectrum. Although the QPSK seems to detect an additional exon at the beginning of the sequence, that shaded area is located far from the true first exon region. Additionally, there are no shaded areas in the region of the last exon. All these results were expected based on the previous analysis of the ROC curve of the methods.

## 6. Conclusions

DNA sequences are symbolic sequences, and, therefore, their numerical representation should not impose additional features on the mapped signal. As seen previously, the spectrum of these signals is sensitive to mapping. That is, for distinct maps, the energy spectrum of a given DNA sequence is also distinct, and they do not represent approximated versions of each other. Furthermore, a fixed mapping must not be able to represent any DNA sequence. Ideally, each DNA sequence must be mapped to a signal using a particular mapping such that this signal captures as much of the information as possible about the sequence. Therefore, in this paper, we propose two algorithms for computing mappings for DNA sequences by using the spectral envelope approach: SNR-SE and TBP-SE.

The proposed algorithms are new methods for finding adaptive complex mappings for DNA sequences, and, hence, improve the spectral analysis of such symbolic sequences. The remarks about the proposed algorithms are summarized as follows. The spectral envelope approach is used to find adaptive mappings and, thus, convert DNA sequences into discrete-time signals. A mapping is uniquely chosen for each sequence according to the constraints: SNR and TBP property. The mapping was defined over a complex field. Both algorithms have loglinear complexity, that is, they are O(NlogN) where *N* is the sequence length. Computational efficiency is essential when large size DNA sequences and databases need to be processed.

To investigate how our algorithms improve the DNA spectral analysis for DNA coding sequence classification, we check the presence or absence of the TBP property at the DNA spectrum for the following methods: Voss [11], EIIP [12], QPSK [13], MEM spectrum [14], SNR-SE and TBP-SE. In this scenario, the proposed method, TBP-SE, had the highest accuracy and sensitivity among all. In addition, the TBP-SE and Voss approaches showed better performance to implement this classification. However, the TBP-SE should be preferred, as it has the highest sensitivity, which is most important in this application since we can reduce the probability of having a coding sequence that will not be identified. We also analyzed the performance of the methods for identifying exonic regions in the gene F56F11.4. In this case, the first exon is the shortest and is usually the most difficult to detect. However, the TBE-SE was the only one to identify the presence of all five exons of the gene.

## Figures and Tables

**Table 1:** CDS identification rate by spectral analysis.

Method	Accuracy (%)	TPR (%)	TNR (%)
Voss [11]	88.51	80.61	96.63
EIIP [12]	86.21	81.77	90.48
QPSK [13]	87.21	78.96	94.77
MEM [14]	76.29	59.43	92.84
SNR-SE	87.43	79.96	94.44
TBP-SE	90.28	89.26	91.74

## Data Availability

Not applicable.
